# *Wild* Bodies Don't Need to Perceive, Detect, Capture, or Create Meaning: They ARE Meaning

**DOI:** 10.3389/fpsyg.2017.01149

**Published:** 2017-07-14

**Authors:** J. Scott Jordan, Vincent T. Cialdella, Alex Dayer, Matthew D. Langley, Zachery Stillman

**Affiliations:** Department of Psychology, Institute for Prospective Cognition, Illinois State University Normal, IL, United States

**Keywords:** theory of mind (ToM), embodiment, embodied cognition, perception, relational properteies, intrinsic properties

For years, experimental psychologists have assumed it is difficult for one person to know the mental states of another because all we can directly experience about each other is observable behavior. As a result, mental states need to be inferred via what has come to be known as a *theory of mind*. According to contemporary *embodiment theorists* however, some of whom refer to themselves as enactivist theorists, the mental states of others are not internally isolated at all, with some arguing social cognition is direct (Gallagher, 2008, 2015) while others propose it can sometimes be constituted by social interaction (De Jaegher et al., 2010).

While we are sympathetic to the complex systems approach embodiment theorists tend to take on the issues of cognition and social interaction, we are concerned their theorizing about *subjective* properties (i.e., *meaning, feelings, experiences*, and *emotions*) leaves such properties vulnerable to epiphenomenalism. That is, the actual *work* of cognition and social interaction is described in terms of complex, multi-scale, causal dynamics among objective phenomena such as neurons, brains, bodies, and worlds, and the *meanings, feelings, experiences*, and *emotions* are said to be emergent from, caused by, identical with, or an informational aspect of, the objective phenomena. In short, the embodiment-driven scientific description of cognition and social interaction renders subjective properties logically unnecessary to the scientific description.

While some embodiment theorists approach the reality of subjective properties via a phenomenological perspective that pretty much assumes the reality of subjective properties without being concerned with potential epiphenomenalism (Gallagher, 2008, 2015; De Jaegher et al., 2010), those who work to establish the non-epiphenomenal reality of experience in a complex systems framework tend to define experience in terms of *relational* properties (Holt et al., 1910; Charles, 2011; Gallagher and Zahavi, 2014; Silberstein and Chemero, 2015), the most popular perhaps being Gibson (1966) and his notion of *affordances*. According to this view, organisms perceive their environment, including other organisms, in terms of behavioral possibilities (i.e., affordances). These possibilities are simultaneously *about* both the organism and the environment. Given they are constituted of bi-directional *aboutness*, they are considered to be inherently *meaningful*. Meaning, in this sense, is being defined in terms of *aboutness*.

The practice of using complex systems theory to describe relational properties has been around for some time (Rosen, 1958; Varela et al., 1991; Kauffman, 1996; Emmeche, 2002). And when we conceptualize relational properties as vehicles of subjective properties via concepts such as affordances, we make good progress toward establishing the non-epiphenomenal status of experience (Silberstein and Chemero, 2015). However, despite the introduction of a relational property (e.g., an affordance) at one level of reality, we leave open the possibility that reality is also constituted of *non-relational* properties; that is, properties that are in no way constituted of their relations with other aspects of reality, what one might refer to as an *intrinsic* property (e.g., weight is a relational property, while mass is an intrinsic property). Such a possibility proves problematic because the notion of intrinsic properties has come under increasing attack by contemporary philosophers of science. According to Jammer (2000), inertial mass emerges from a particle's interaction with the Higgs field: “…a scalar field that ‘permeates all of space’ and ‘endows particles with mass’ (p. 162).” Bauer (2011) asserts this type of interactive dependence renders mass *externally grounded*, which means the particle's mass is partially constituted by its relations to its context. Others have rendered similar criticisms of the notion of intrinsic properties via concepts such as *ultra-grounding* (Harré, 1986) and *Global Groundedness* (Prior et al., 1982). In a similar vein, Schaffer (2003) and Dehmelt (1989) claim that there may no fundamental level to reality at all.

Such an assault on intrinsic properties challenges the idea that some properties are relational and others are not which, in turn, problematizes the idea of defining one level of reality (i.e., the internal dynamics of a single-cell, or an organism-environment coordination) as being meaningful because it entails a relational property. According to Wild Systems Theory (WST—Jordan and Day, 2015), *all* properties are constituted *of* and *by* their relations with context. As a result, all properties are inherently meaningful because they are naturally and necessarily *about* the contexts within which they persist. From this perspective, meaning is ubiquitous. In short, reality is inherently meaningful.

Given this notion of an inherently relational, meaningful reality, WST goes beyond the notion of affordances and proposes instead that organisms *are* meaning because they are inherently relational in that they constitute embodiments of the constraints (i.e., contexts) they have had to phylogenetically, as well as ontogenetically embody in order to sustain themselves (Jordan and Ghin, 2006, 2007). Bones, muscles, and brains for example, constitute embodiments of the constraints involved in propelling a body as a whole, through a gravity field. At every level of scale, from the single-cell up through the organism-environment coordination, such *wild* bodies are inherently relational and, therefore, inherently meaningful (Streeck and Jordan, 2009). As a result, *wild* bodies are not *information detectors* or *information processors*, but rather, *modulators of context*.

WST's ontology of ubiquitous, multi-scale relationality firmly establishes the reality of subjective properties by revealing the intrinsic-relational dualism that lies at the heart of most contemporary takes on relational properties. If reality is inherently relational, all the way down, we do not need to posit vehicles of content. And given that *other* organisms were part of the contextual constraints that organisms had to embody to sustain themselves, social interaction is only special in that it constitutes yet another level of the inherently meaningful, relationality in which all *wild* bodies are nested.

To be sure, some may feel that by making *meaning* ubiquitous, WST ultimately renders it meaningless. Jordan and Day (2015) propose however, that because everything is meaningful, *nothing* is meaningless. Jordan and Vinson (2012) propose that non-living systems also constitute embodiments of context and, as a result, are also inherently meaningful (i.e., inherently *about* the contexts they embody). What distinguishes the aboutness entailed in living and non-living systems is the dynamics by which such systems sustain their integrity. Non-living systems exist as “systems” in a persistent state of tension between strong and weak forces, and their micro-macro structures are not coupled in ways that sustain any particular aspect of the coupling in response to changes in these forces. The micro-macro dynamics of living systems however (e.g., the chemicals that constitute a single cell, and the cell as a whole, respectively), are dynamically coupled in ways that generate work (i.e., energy transformations) that continually bring energy into the system and allow it to generate and sustain ordered states (e.g., organelle maintenance, genetic transcription, and the Krebs cycle) capable of resisting, to some extent, the strong and weak forces within which such systems are perpetually nested. Regardless of the dynamical differences between living and non-living systems however, both constitute embodiments of context and, as a result are inherently relational and meaningful. From this perspective, phenomena such self-awareness, qualia, and consciousness are phylogenetically scaled-up recursions of the meaning inherent in all embodiments of context. In short, one might regard phylogenetic history as the evolution of meaning.

In conclusion, it is perhaps a bit unfair to hold embodiment theorists responsible for overcoming epiphenomenalism. Cognitive science as a whole has been working to ground experience and subjectivity for quite some time. Much to their credit, contemporary enactivists pay close attention to phenomenology and develop research methods that include phenomenology as an important aspect of the research. And according to WST, this extremely valuable research will definitely advance our understanding of the relations that exist between brains, bodies, environments, and phenomenology. In the end however, such research will not prove necessary to grounding phenomena we refer to as “experience” and “subjectivity” because such phenomena are phylogenetically scaled-up versions of the same inherent relationality that constitutes all phenomena. Human *consciousness*, human *subjectivity*, and human *meaning* constitute evolved forms of inherent relationality—evolved forms of meaning. In essence, one might say that *meaning* is reality interacting with itself.

## Author contributions

All authors listed have made a substantial, direct, and intellectual contribution to the work, and approved it for publication.

### Conflict of interest statement

The authors declare that the research was conducted in the absence of any commercial or financial relationships that could be construed as a potential conflict of interest.
